# Birth outcome in women with breast cancer

**DOI:** 10.1038/sj.bjc.6602878

**Published:** 2005-11-22

**Authors:** V Langagergaard, M Gislum, M V Skriver, B Nørgård, T L Lash, K J Rothman, H T Sørensen

**Affiliations:** 1 Department of Clinical Epidemiology, Aarhus University Hospital, Ole Worms Allé 150, DK-8000, Aarhus C, Denmark; 2 Department of Epidemiology, Institute of Public Health, Aarhus University, Vennelyst Boulevard 6, DK-8000, Aarhus C, Denmark; 3 Department of Epidemiology, School of Public Health, Boston University, 715 Albany Street, TE3, Boston, MA 02118, USA

**Keywords:** epidemiology, breast cancer, birth outcome, cohort study

## Abstract

We investigated whether maternal breast cancer affects birth outcome in a nationwide cohort study of 695 births from 1973 to 2002 of women with breast cancer with respect to preterm birth, low birth weight at term, stillbirth and congenital abnormalities as well as mean birth weight, compared with the outcomes of 33 443 births from unaffected mothers. There was no excess risk of adverse birth outcome for the 216 newborns of women with breast cancer before pregnancy. Stratification by mother's treatment did not change the results. For 37 newborns of women diagnosed during pregnancy, the prevalence ratio (PR) of preterm birth was 8.1 (95% confidence interval (CI): 3.8–17). However, 10 of the 12 preterm deliveries among these women were elective early deliveries. Among 442 births of women diagnosed in the 2 years from time of delivery, the PR of preterm birth was 1.4 (95% CI: 1.0–2.0), and the PR of low birth weight at term for boys was 2.9 (95% CI: 1.3–6.3). Overall, our results are reassuring regarding the risks of adverse birth outcome for breast cancer patients.

In western countries many women postpone childbearing for personal or professional reasons (Dow *et al*, 1994), which both increases their risk for breast cancer (Kelsey *et al*, 1993) and reduces the period between giving birth and breast cancer diagnosis. In the future, therefore, more breast cancer patients will have recently given birth, been pregnant concurrent with their diagnosis, or not yet started their childbearing at the time of their diagnosis.

Biological mechanisms related to the cancer or its treatment may impact foetal growth, development, and teratogenesis (Zemlickis *et al*, 1992; Zhu *et al*, 2002). However, the epidemiologic evidence of the effect of breast cancer on birth outcome is limited. The few studies of women who were diagnosed with breast cancer before pregnancy have focused on maternal prognosis (Ribeiro *et al*, 1986; Sutton *et al*, 1990; Dow *et al*, 1994; Malamos *et al*, 1996; Kroman *et al*, 1997; Velentgas *et al*, 1999; Blakely *et al*, 2003). Thus, no population-based cohort study of birth outcome in women diagnosed with breast cancer before pregnancy has been published. Cohorts without control groups including between four and 121 women (Ribeiro *et al*, 1986; Daly and Donnellan, 1992; Berry *et al*, 1999; Giacalone *et al*, 1999; Kuerer *et al*, 2002; Ring *et al*, 2005) have shown that the majority of women who are diagnosed with breast cancer during or shortly after pregnancy give birth to healthy children. Two controlled studies, however, suggested an increased risk of preterm birth and low birth weight for offspring of these women (Zemlickis *et al*, 1992; Smith *et al*, 2001). Therefore, within a cohort study, we examined birth outcome in all women diagnosed with breast cancer in Denmark from 1943 to 2002, compared with women without cancer.

## MATERIAL AND METHODS

### Study population

We conducted this nationwide cohort study based on all Danish women who were diagnosed with breast cancer from January 1, 1943 through December 31, 2002, and who gave birth from January 1, 1973 through December 31, 2002. Women were included if they were diagnosed at any time before pregnancy, during the pregnancy, or until 2 years post partum. Their birth outcome was compared with the outcome in a comparison cohort selected from other births in Denmark. We restricted all analyses to singleton births only, and each pregnancy was included in the analyses as an independent event.

#### Breast cancer cohort

Women with breast cancer were identified from the Danish Cancer Registry, which has kept records of all incident cases of cancer in Denmark since 1943, classified according to the International Classification of Diseases (ICD-7) (Storm *et al*, 1997). The records include the civil registration number, diagnosis, date of diagnosis, method of verification, extent of spread of the tumour at time of diagnosis, and treatment given within 4 months after diagnosis. We identified all women with a diagnosis of breast cancer (ICD-7 codes 170.0–170.5). We excluded all cases of ‘Carcinoma *in situ*’ and six cases of sarcoma involving the breast. Women with breast cancer were linked to the Danish Medical Birth Registry with data on all births in Denmark since January 1, 1973 (Kristensen *et al*, 1996) obtained from birth notifications, filled in by midwives (in Denmark all births, including home births, are attended by midwives). The main variables are the civil registration number of the mother and child, date and place of birth, gestational age, birth weight, and parity. Birth weights ⩾7000 g probably reflected coding errors and were excluded from the analyses. Likewise, we excluded births registered without a gestational age or when this was less than 20 weeks or more than 44 weeks. Owing to a change in classification procedures in the Birth Registry in 1978, there was more missing data on gestational age for the years 1978–1981 than for other years (mean missing proportion, 21.7% for 1978–1981, compared with 2.4% for the years 1973–1977, and 0.7% for the years 1982–2002). We identified 695 singleton births delivered by women in the breast cancer cohort.

#### Comparison cohort

For each birth by a woman with breast cancer, 50 comparison births matched by month and year of birth, by county of mother's residence, and born to 50 different women, who were not diagnosed with any cancer before or during the pregnancy or until 2 years after the birth were selected from the Birth Registry. If fewer than 50 comparison births fulfilled the criteria, we used the available number of births. If more than 50 comparison births were eligible after matching, the subset of 50 was randomly selected. On average, 48 comparison births were selected for each exposed birth. Altogether, 33 443 single births were selected for the comparison cohort.

#### Outcome data

The data collected from the Birth Registry included preterm birth (birth before 37 completed weeks of pregnancy), low birth weight at term (birth weight <2500 g with a gestational age ⩾37 completed weeks of pregnancy), stillbirth (delivery of a dead foetus at 28 completed weeks of gestation or later in pregnancy), male proportion of newborns, and birth weight. Data on potential confounders included maternal age, parity, gestational age, and calendar period of the birth. Data about congenital abnormalities (including chromosomal abnormalities) diagnosed during the first year after the birth were collected from the Danish Hospital Discharge Registry, established in 1977, with records of all discharges from Danish hospitals. The recorded information includes the civil registration number, dates of admission and discharge, and up to 20 discharge diagnoses, using the International Classification of Diseases (ICD-8 before 1994 and ICD-10 from 1994 onward (Andersen *et al*, 1999)). The codes for congenital abnormalities (including chromosomal abnormalities) were 740.00–759.99 in ICD-8 and Q0.00–Q99.9 in ICD-10. Diagnoses of congenital dislocation of the hip and undescended testis were excluded because of their poor validity (Larsen *et al*, 2003).

### Record linkage

Linkage between registries was made by the civil registration number stored in the Danish Civil Registration System together with information on vital status, emigration, address, and nuclear family members' civil registration number since 1968 (Frank, 2000).

### Data analysis

We classified the births of women with breast cancer according to time of cancer diagnosis in relation to pregnancy. **Group 1** included the first birth after breast cancer delivered by women who were diagnosed at any time before pregnancy. **Group 2** included the births delivered by women with a diagnosis of breast cancer during their pregnancy (i.e., diagnosed between the first day in the last menstruation until the date of birth). **Group 3** included births delivered by women who were diagnosed with breast cancer after delivery (i.e., diagnosed between the day after the date of birth until 2 years later). If a woman gave birth more than once in this 2-year period, only the last birth before the cancer diagnosis was included. We computed the difference between the male proportion of newborns of women with breast cancer and that of newborns of matched comparison mothers with 95% confidence intervals (95% CI) for these differences. We estimated the prevalence ratios (PR) using prevalence odds ratios and 95% CI for preterm birth, low birth weight at term, stillbirth, and congenital abnormalities by logistic regression modelling. Stillborn children were excluded from the analyses of preterm birth, low birth weight at term, and congenital abnormalities. We adjusted for maternal age, parity, and calendar period of the birth. PRs for congenital abnormalities were additionally adjusted for gestational age. For births in Groups 1 and 3, we repeated the analyses in strata of boys and girls to examine if sex of the child modified the PR estimates. For births in Group 1, we evaluated whether treatment of the mother modified the PR estimates by repeating the analyses in strata of births of women treated with surgery alone and births of women who received other treatment (i.e., radiotherapy, chemotherapy, or endocrine therapy).

We used multivariate regression analysis to estimate differences in mean birth weight adjusted for maternal age, parity, gestational age, and calendar period of the birth. Stillborn children were excluded from this analysis.

All analyses used SAS software, version 8.2.

The study was approved by the Danish Data Protection Agency (record no. 2003-41-2833).

### RESULTS

Descriptive data on Groups 1, 2 and 3 and their matched comparison births are shown in Table 1. Of the 695 single births delivered by women with BC, 216 occurred in Group 1, 37 occurred in Group 2, and 442 occurred in Group 3. For Group 1, the median number of days from the time of diagnosis until pregnancy (i.e., the first day in the last menstruation) was 753 days (range: 3–5965 days). Of the 37 births in Group 2, one woman was diagnosed in the first trimester, five in the second, and 31 women were diagnosed in the third.

For births delivered by women in Group 3, the median number of days from date of the birth until date of cancer diagnosis was 417 days (range: 1–729 days).

We evaluated the proportion of male newborns of women with breast cancer compared with that of newborns of unaffected mothers (50 *vs* 52%, difference=−2.2%, (95% CI=−8.9; 4.5) for Group 1, 49 *vs* 52%, difference=−3.4%, (95% CI=−20; 13) for Group 2, and 53 *vs* 51%, difference=2.5%, (95% CI=−2.2; 7.2) for Group 3).

Table 2 shows the PRs for preterm birth, low birth weight at term, stillbirth and congenital abnormalities for newborns in Groups 1–3. There was no stillborn child among the births delivered by mothers with breast cancer. For births in Group 1, we found no increased odds of low birth weight at term or congenital abnormalities and no substantial increased odds of preterm birth. For births in Group 2, the odds of preterm birth increased by eight-fold (PR=8.1, 95% CI=3.8–17). However, 10 of the 12 preterm deliveries among the women with breast cancer were elective preterm deliveries. As a result of the small number of outcome events, effect estimates for Group 2 were imprecise. For Group 3 the PR of preterm birth was 1.4 (95% CI=1.0–2.0). For low birth weight at term the PR was 1.4 (95% CI=0.7–2.8). There was no increased prevalence of congenital abnormalities. We found no clusters of congenital abnormalities in any specific organ system and there was only one case with a chromosomal abnormality (data not shown). Stratification according to sex of the offspring in Groups 1 and 3 did not change the overall effect estimates substantially (data not shown), except for low birth weight at term in Group 3, in which boys had almost three-fold increased odds (PR=2.9; 95% CI: 1.3–6.3), and girls had decreased odds (PR= 0.3; 95% CI: 0.03–2.0). For births in Group 1, stratification according to mother's treatment (surgery alone or other treatment) did not change the overall results (data not shown).

Table 3 shows the adjusted mean differences in birth weight between babies born to women with breast cancer and babies born to comparison mothers. Newborns of women in Groups 1 and 3 had nearly the same mean birth weights as newborns of comparison mothers, whereas newborns of women in Group 2 had a mean birth weight 240 g (95% CI=−404; −76) less than newborns of comparison mothers.

## DISCUSSION

We examined the association between maternal breast cancer and adverse birth outcome in a nationwide cohort and found little difference in the occurrence of preterm birth, low birth weight at term, stillbirth, or congenital abnormalities, compared with the comparison cohort, among newborns of women who were diagnosed with breast cancer before pregnancy.

The eight-fold increased odds of preterm birth for newborns of women who were diagnosed with breast cancer during their pregnancy reflected a higher rate of elective early delivery, probably to allow an earlier start of cancer therapy. After adjustment for gestational age, there was a 240 g reduction in mean birth weight for newborns in this group. The association with preterm birth in Group 3 may be explained by suboptimal intrauterine conditions caused by a preclinical cancer. In this group, only boys had increased odds of low birth weight at term, suggesting that male foetuses are more vulnerable than female.

Our data are derived from a uniformly organized health care system with complete cancer and birth registration. Some selection problems are possible, however. If women with breast cancer had more miscarriages or induced abortions caused by foetal abnormalities than comparison mothers, this phenomenon could explain why we found no increased risk of congenital abnormalities. It has been suggested that exposure to severe periconceptional life events might reduce the male proportion of offspring, partly because of differential abortion of male embryos (Hansen *et al*, 1999). Thus, a lower proportion of males for offspring of the patients could be an indicator of miscarriages. Another study has indicated an increased risk of miscarriage among women with breast cancer (Velentgas *et al*, 1999). Our data, however, did not show any important difference in male proportions between the offspring of breast cancer women and offspring of comparison mothers. It has been reported that women with high socioeconomic status have a higher incidence of breast cancer (Danø *et al*, 2004), while low socioeconomic status has been associated with adverse birth outcome (Luo *et al*, 2004). We were unable to adjust for socioeconomic status and therefore we may have underestimated the effect of the disease.

A recent study found that treatment data recorded in the Cancer Register are of varying quality (Jensen *et al*, 2002). However, breast cancer treatment with surgery alone was correctly registered for 95.4% (Jensen *et al*, 2002). Coding mistakes are infrequent in the Birth Registry, but data have some misclassifications of gestational age (Kristensen *et al*, 1996). Our data did not suggest any differential misclassification of preterm birth between women with breast cancer and comparison mothers.

Hospital discharge data are not always coded correctly (Larsen *et al*, 2003), but Danish data on congenital abnormalities are of high quality compared with other countries, with 80–85% coded correctly (Larsen *et al*, 2003). We did not find any clusters of congenital abnormalities in any specific organ system.

Our finding of an increased risk of giving birth preterm for women who were diagnosed with breast cancer during or shortly after pregnancy corroborates the results of two earlier studies (Zemlickis *et al*, 1992; Smith *et al*, 2001). In a hospital-based study, Smith *et al* (2001) identified 423 cases of breast cancer diagnosed from 9 months preceding delivery until 12 months after delivery over a period of 6 years in California. They reported an odds ratio of 2.2 (95% CI=1.7–2.8) for prematurity, and an odds ratio of 2.0 (95% CI=1.0–4.1) for very low birth weight. They adjusted only for maternal age. A hospital-based historical cohort study from 1992 of 118 women, who were pregnant within 9 months before or 3 months after their first treatment for breast cancer, reported a lower mean birth weight after adjustment for gestational age and a higher proportion of preterm births among offspring of women with breast cancer compared with controls (Zemlickis *et al*, 1992). In these studies, however, the authors did not distinguish between birth outcome of women diagnosed with breast cancer during their pregnancy and women diagnosed shortly after pregnancy. We found a lower mean birth weight limited to newborns of women diagnosed during their pregnancy.

In conclusion, this is the first population-based cohort study of birth outcome in women diagnosed with breast cancer before pregnancy, and the largest cohort study to date of birth outcome in women diagnosed with breast cancer during or shortly after pregnancy. Overall, our results are reassuring regarding the risks of adverse birth outcome for women with breast cancer.

## Figures and Tables

**Table 1:** Characteristics of births of women with breast cancer and of the comparison cohort

	**Births in group 1 (*N*=216)**	**Births in comparison cohort (*N*=10 453)**	**Births in group 2 (*N*=37)**	**Births in comparison cohort (*N*=1795)**	**Births in group 3 (*N*=442)**	**Births in comparison cohort (*N*=21 195)**
*Maternal age at delivery, number (%)*						
<25 years	5 (2.3)	2441 (23.4)	1 (2.7)	419 (23)	18 (4.1)	5517 (26.0)
25–29 years	29 (13)	4054 (38.8)	8 (22)	693 (39)	104 (24)	8256 (39.0)
30–34 years	76 (35)	2853 (27.3)	13 (35)	486 (27)	184 (42)	5383 (25.4)
⩾35 years	106 (49)	1105 (10.6)	15 (41)	197 (11)	136 (31)	2039 (9.6)
Data missing	0 (0.0)	0 (0.0)	0 (0.0)	0 (0.0)	0 (0.0)	0 (0.0)
						
*Age at delivery (years)*						
Mean (±s.d.)	34.4 (±4.8)	28.2 (±4.9)	33.3 (±4.8)	28.3 (±5.0)	32.2 (±4.4)	27.8 (±4.9)
Min/max	21–46	15–50	24–44	15–43	20–44	14–47
						
*Parity, number (%)*						
1	92 (43)	4778 (45.8)	11 (30)	849 (47)	116 (26)	9514 (44.9)
⩾2	124 (57)	5665 (54.2)	26 (70)	946 (53)	326 (74)	11 665 (55.1)
Data missing	0 (0.0)	10 (<0.1)	0 (0.0)	0 (0.0)	0 (0.0)	16 (<0.1)
						
*Offspring (sex), number (%)*						
Male	108 (50)	5454 (52.2)	18 (49)	934 (52)	236 (53)	10 782 (50.9)
Female	108 (50)	4989 (47.8)	19 (51)	861 (48)	206 (47)	10 397 (49.1)
Data missing	0 (0.0)	10 (<0.1)	0 (0.0)	0 (0.0)	0 (0.0)	16 (<0.1)
						
*Gestational age (weeks)* a						
Mean (±s.d.)	39.2 (±2.4)	39.6 (±1.8)	37.2 (±3.8)	39.5 (±1.9)	39.3 (±2.0)	39.6 (±1.9)
Min/max	25–43	20–44	24–42	26–43	25–43	23–44

a Stillborn babies were excluded from the analyses of mean gestational age.

Group 1: Births of women diagnosed with breast cancer before pregnancy.

Group 2: Births of women diagnosed with breast cancer during pregnancy.

Group 3: Births of women diagnosed with breast cancer from the day after giving birth and until 2 years later.

**Table 2:** Crude and adjusted prevalence odds ratios of birth outcome in women with breast cancer

	**Breast cancer cohort outcome/total (%)**	**Comparison cohort outcome/total (%)**	**Crude prevalence odds ratio (95 % ci)**	**Adjusted prevalence odds ratio^a^ (95 % CI)**
*Births in group 1*	(*N*=216)	(*N*=10 453)		
Preterm birth^b^	14/216 (6.5)	507/10 414 (4.9)	1.4 (0.8–2.3)	1.3 (0.7–2.2)
Low birth weight^b^	3/202 (1.5)	137/9885 (1.4)	1.1 (0.3–3.4)	1.2 (0.4–3.8)
Stillbirth	0/216 (0.0)	39/10 453 (0.4)	—	—
Abnormalities^b^,^c^	7/203 (3.4)	369/9775 (3.8)	0.9 (0.4–1.9)	0.9 (0.4–1.9)
				
*Births in group 2*	(*N*=37)	(*N*=1795)		
Pretem birth^b^	12/37 (32)	102/1785 (5.7)	7.9 (3.9–16)	8.1 (3.8–17)
Low birth weight^b^	1/25 (4.0)	19/1679 (1.1)	3.6 (0.5–28)	5.3 (0.6–51)
Stillbirth	0/37 (0.0)	10/1795 (0.6)	—	—
Abnormalities^b^,^c^	1/35 (2.9)	53/1685 (3.1)	0.9 (0.1–6.7)	0.5 (0.1- 3.6)
				
*Births in group 3*	(*N*=442)	(*N*=21 195)		
Pretem birth^b^	33/442 (7.5)	1143/21 120 (5.4)	1.4 (1.0–2.0)	1.4 (1.0–2.0)
Low birth weight^b^	9/408 (2.2)	329/19 917 (1.7)	1.3 (0.7–2.6)	1.4 (0.7–2.8)
Stillbirth	0/442 (0.0)	75/21 195 (0.4)	—	—
Abnormalities^b^,^c^	16/389 (4.1)	685/18 519 (3.7)	1.1 (0.7–1.9)	1.1 (0.6–1.8)

Group 1: Birth outcome in women diagnosed with breast cancer before pregnancy.

Group 2: Birth outcome in women diagnosed with breast cancer during pregnancy.

Group 3: Birth outcome in women diagnosed with breast cancer from the day after giving birth and until 2 years post partum.

a Prevalence odds ratios for preterm birth and low birth weight at term were adjusted for maternal age (<25 year, 25–29 year, 30–34 year and ⩾35 year), parity (1, 2+) and calendar period of birth (73–86, 87–94, 95–02). Prevalence odds ratios for congenital abnormalities were additionally adjusted for gestational age (20–33 week, 34–36 week and ⩾37 week).

b Stillborn babies were excluded from the analyses of preterm birth, low birth weight at term and congenital abnormalities.

c Data on congenital abnormalities included births from 1977 to 2002.

**Table 3:** Mean birth weight for newborns of women with breast cancer and for the comparison cohort

	**Breast cancer**		**Comparison cohort**		**Mean difference in birth weight (g)**	
		**Mean birth weight (g)**		**Mean birth weight (g)**	**Crude**	**Adjusted (95% confidence limits)^a^,^b^**
*Births in group 1*	*N*=216	3411	*N*=10 388	3474	−63	−54 (−122; 13)
Data missing	0		26			
						
*Births in group 2*	*N*=37	2948	*N*=1781	3472	−524	−240 (−404; −76)
Data missing	0		4			
						
*Births in group 3*	*N*=441	3471	*N*=21 054	3466	5	−5 (−52; 42)
Data missing	1		66			

Group 1: Mean birth weight for newborns of women with breast cancer before pregnancy.

Group 2: Mean birth weight for newborns of women diagnosed with breast cancer during pregnancy.

Group 3: Mean birth weight for newborns of women with breast cancer from the day after giving birth until 2 years post partum.

a Adjusted for gestational age (20–33 week, 34–36 week and ⩾37 week), mother's age (<25 year, 25–29 year, 30–34 year, ⩾35 year), parity (1, 2+) and calendar period for birth (73–86, 87–94, 95–02) in a multivariate regression model.

b Stillborn babies were excluded from the analysis.
